# Effects of Short-Term Treatment of Hanwoo Satellite Cells with Various Concentrations of Cortisol

**DOI:** 10.3390/ani15192847

**Published:** 2025-09-29

**Authors:** Leecheon Kim, Dongjin Yu, Hyunwoo Choi, Jongryun Kim, Junseok Ban, Kwanseob Shim, Darae Kang

**Affiliations:** 1 Department of Animal Resources and Biotechnology, College of Agriculture Life Science, Jeonbuk National University, Jeonju 54896, Republic of Korea; rladlcjs4483@naver.com (L.K.); panda8028@jbnu.ac.kr (D.Y.); dahaseyo@naver.com (J.K.); bankingban@jbnu.ac.kr (J.B.); 2 Department of Agricultural Convergence Technology, Jeonbuk National University, Jeonju 54896, Republic of Korea; choihw@jbnu.ac.kr (H.C.); ksshim@jbnu.ac.kr (K.S.); 3 Department of Animal Science, Jeonbuk National University, Jeonju 54896, Republic of Korea; 4 Department of Animal Biotechnology, Jeonbuk National University, Jeonju 54896, Republic of Korea; 5 Institute of Agricultural Science and Technology, Jeonju 54896, Republic of Korea

**Keywords:** cortisol, Hanwoo, short-term stress, muscle stem cell, cellular viability

## Abstract

**Simple Summary:**

Stress in livestock is inevitable and one of the factors that decrease productivity. In this study, the effect of cortisol on Korean native cattle (Hanwoo) muscle stem cells (HWSCs) under short-term stress was evaluated. No negative effects were observed in terms of antioxidant factors, cell death, or cellular respiration. The specific responses observed in livestock muscle cells in this study can be used as biological indicators to indirectly assess the stress experienced by livestock. These findings provide valuable basic data for developing effective methods to manage and control stress in livestock.

**Abstract:**

Transportation, environmental changes, and overcrowding can induce short-term stress in livestock, leading to physiological imbalances even within a short period. Cortisol is a stress-response hormone and its concentration in the blood can rapidly fluctuate depending on the individual and situation. This study evaluated the short-term effects of cortisol by applying blood cortisol concentrations that mimicked the normal and stress-induced levels observed in Korean native cattle (Hanwoo) to the culture medium of Hanwoo muscle stem cells (HWSC). Treatments were designed with five cortisol concentrations (0, 5, 10, 30, and 70 ng/mL) and four treatment times (0.5, 1, 2, and 3 h), based on the CCK-8 and viable cell count results. The expression levels of cortisol receptor-related genes (*NR3C1*, *HSP70*, and *HSP90AA1*) increased and reached a peak at 30 min post-treatment. After 30 min, the expression of these genes gradually decreased. However, in the case of *HSP70*, expression tended to increase again after 3 h of treatment. This could be seen as the regulation of cortisol inflow into the HWSC. Upon examining the oxidative effects of cortisol on superoxide dismutase 1 (*SOD1*), glutathione peroxidase (*GPX*), catalase (*CAT*), and oxygen consumption rate (OCR), the expression of antioxidant factors increased and peaked at 30 min of treatment. Following this peak, their levels generally began to decrease. However, in the 70 ng/mL group, the expression of these factors remained at a high level compared to the control group even after 30 min. In addition, the cellular respiration index and ATP production increased as the treatment prolonged, regardless of the concentration, as shown by the OCR analysis. These results can be considered a phenomenon corresponding to the accumulation of oxidative by products, such as Reactive Oxygen Species (ROS), caused by cortisol. The gene expression of apoptosis factors (*p53*, *BAX*, *Caspase-3*) temporarily increased at 30 min but then decreased. Caspase-3 protein activity was elevated at 30 min in the 70 ng/mL group, which later reduced. These results suggested that short-term cortisol administration had no effect on apoptosis in muscle cell culture. Therefore, the study findings elucidating the effects of short-term cortisol treatment on HWSC suggest that short-term stress may not have a significant negative effect on Hanwoo muscle. However, as this study was limited to muscle stem cells derived from Hanwoo, further investigation is required to determine whether the observed responses are consistent across different species and in vivo environments.

## 1. Introduction

Stress is inevitable during livestock production. It can be caused by various factors, including environmental, psychological, and metabolic conditions. Transport stress is short-term stress that induces physiological changes as an adaptive response [1]. Cortisol, a hormone that corresponds to external/internal physical and mental stress, is regulated by the hypothalamus–pituitary–adrenal axis (HPA) and the major glucocorticoids synthesized in the adrenal cortex [2]. However, the concentration of cortisol in the blood varies among individuals. The cortisol level in cattle is 15–25 nmol/L under normal conditions and increases rapidly to 60–200 nmol/L depending on the level of stress [3].

Cortisol is a major stress hormone that significantly affects livestock tissue function. Studies on its effects in various animal species are actively being conducted. Bomfim et al. [4] investigated the effects of cortisol on milk production and mammary epithelial cell viability in Saanen goats. When cortisol levels are low, the gene expression of growth hormone receptor (*GHR*) and prolactin receptor (*PRLR*) increases, resulting in higher milk production. Conversely, treatment of mammary epithelial cells with high cortisol levels led to increased expression of the apoptotic gene *BAX*, whereas the expression of genes related to cell survival, such as insulin receptor (*INSR*) and insulin-like growth factor 1 receptor (*IGF1R*), decreased. These findings suggest that while an appropriate concentration of cortisol may enhance mammary cell function, excessive cortisol levels can induce apoptosis and negatively affect cell viability.

Similarly, studies by Dong et al. [5,6] examined the effects of cortisol on bovine endometrial epithelial cells. When these cells were treated with various concentrations of cortisol, the mRNA expression of inflammatory mediators and cytokines was downregulated. Treatment with 5 ng/mL (basal physiological level), 15 ng/mL (physiological level during parturition), and 30 ng/mL (levels exceeding the physiological range owing to exogenous administration or pathological states) for 0, 3, 12, and 18 h revealed that cell proliferation was the highest at 15 ng/mL. This indicates that elevated cortisol levels after parturition promote tissue recovery by stimulating endometrial epithelial cell proliferation. Siddiqui et al. [7] demonstrated that cortisol reduces the viability of porcine muscle stem cells and fibroblasts in both monoculture and coculture systems, indicating that cortisol may impair muscle growth and recovery. Fast et al. [8] investigated cortisol response and immune effects under short- and long-term stress in brown trout. Their findings showed that cortisol affects the expression of immune factor *IL-1β*, indicating that its expression can be regulated by stress. Studies with various animal species have shown that cortisol may have a dual effect on tissue function and viability, with positive and negative results depending on the concentration and treatment time.

However, investigating the short-term effects of cortisol in vivo in large livestock, such as cattle and pigs, is economically limited and there are few studies on the effects of cortisol over a very short period in skeletal muscle tissue and cells.

This study aimed to investigate the short-term effects of cortisol on muscle stem cells derived from Hanwoo. By treating cells with cortisol concentrations corresponding to normal conditions and stress-induced levels observed in vivo, we evaluated the impact on cellular viability. The findings of this study contribute to a better understanding of tissue-specific stress responses in livestock and provide fundamental data for improving stress management and productivity in Hanwoo production systems.

## 2. Materials and Methods

### 2.1. Cell Culture

Hanwoo muscle stem cells (HWSCs) were provided by the Generative Engineering Laboratory of Jeonbuk National University [9]. The HWSCs were grown in a growth medium consisting of 83% Ham’s F-10 (11550043, Gibco, Grand Island, NE, USA), 15% Fetal bovine serum (10270106, Gibco), 1% L-glutamine (25030081, Gibco), and 1% Penicillin-Streptomycin (15140122, Gibco), and the third-to-fourth passage cultured (P3, P4) cells were used in the experiment. The treatments were divided according to the cortisol concentration (C-106-1ML, Cerilliant, Round Rock, TX, USA) treatment (0, 5, 10, 20, 30, 50, and 70 ng/mL), and treatment time (15 min, 30 min, 1 h, 2 h, 3 h, and 24 h). All analyses were performed in at least triplicate to ensure technical reliability.

### 2.2. Selection of Cortisol Concentration and Treatment Time

CCK-8 (CK04-11, Dojindo, Kumamoto, Japan) analysis and live cell counting was used to determine the cortisol concentration and treatment time. HWSCs were seeded at a density of 1 × 104 cells/well (P3) in a 96-well plate and an additional culture was performed for 24 h for cell adhesion. After adding the culture medium with various concentrations of cortisol, the cells were cultured for the designated treatment times. After cortisol treatment, 10 μL CCK-8 reagent was added to each well and additional culture was conducted for 1 h in a 5% CO_2_ incubator. For analysis, 450 nm absorbance analysis was performed using a Microplate Reader (N10588, Thermo Fisher Scientific, Waltham, MA, USA) according to the manufacturer’s instructions. During the CCK-8 assay (Table 1), cortisol caused a significant decrease in cell viability at 15 min, a significant increase at 30 min regardless of concentration, and a significant decrease with increasing time from 1 h (*p* < 0.05). Based on these results, considering the objective of this study to observe short-term stress responses and the physiological concentration range used in previous studies, the long-term treatment conditions of 24 h and specific concentrations (20, 50 ng/mL) were excluded from the live cell count analysis.

For live cell count analysis, HWSCs were seeded at a density of 2.4 × 10^5^ cells/well (P3) in a 6-well plate and then cultured for 72 h. The experiment was conducted for each treatment time after replacing the medium with five types of culture media mixed with different concentration of cortisol. HWSCs from all treatments were washed twice with DPBS (14190136, Gibco) and separated using 0.25% Trypsin EDTA (25200072, Gibco). Isolated HWSC counting was analyzed using the Acridine Orange/Propidium Ideal Stain (F23001, Logos Biosystem, Anyang, Republic of Korea) according to manufacturing guidelines and analyzed with LUNA-FL™ Dual Fluorescence Cell Counter (L20001, Logos Biosystem). The number of live HWSCs was calculated relative to that in the control group for each treatment time. During the live cell count analysis (Table 2), cell viability significantly decreased at 15 min, and either increased or decreased over time (*p* < 0.05). Although the overall results from the CCK-8 and live cell count analysis differed, the 15 min treatment was excluded from subsequent analyses because a consistent, significant decrease was observed in both methods.

### 2.3. RNA and Protein Extraction

HWSCs for RNA and protein extraction were seeded with 2.0 × 10^5^ cells/well (P3) in a 10 cm dish and then incubated in the same manner as conducted during the viable cell count analysis. RNA extraction was performed using AccuPrep Universal RNA Extraction Kit (K-3140, BIONEER, Daejeon, Republic of Korea) according to the manufacturer’s guidelines and RNA concentration and purity were analyzed at 260 nm and 260 nm/280 nm using a microvolume spectrophotometer (K12C-KR-001W, KLAB, Namyangju-si, Republic of Korea), respectively. Protein extraction was performed by adding RIPA buffer (RC2002-050-00, Biosang, Yongin-si, Republic of Korea) with Protease inhibitor (A32963, Invitrogen, Grand Islasd, NE, USA) to the cell pellet, performing an incubation with interval vortex mix, and centrifuging (15,000 rpm, 30 min, 4 °C). Protein concentrations were measured using a DC protein assay kit (5000113, 5000114, 5000115, Bio Rad, Hercules, CA, USA) and protein concentrations (1 μg protein/μL) in each sample were adjusted using a loading buffer (161-0737, Bio Rad) mixed with β-mercaptoethanol (1610710, Bio Rad). The prepared sample was heated in a 95 °C heating block for 5 min and then used in the experiment.

### 2.4. RT-qPCR

cDNA was synthesized using 1 μg of RNA subjected to reverse transcription with AccuPower CycleScript RT PreMix (K-2047-B, BIONEER) according to manufacturer’s guidelines. Synthesized cDNA was amplified using AccuPower 2x GreenStar qPCR Master Mix (K-6253, BIONEER) in a CFX96 Duet Real-Time PCR System (12016265, Bio Rad). Each gene primer sequence (Table 3) was generated using the NCBI Primer-BLAST tool (https://www.ncbi.nlm.nih.gov/tools/primer-blast/, accessed on 2 August 2024). The amplification was repeated 40 times, starting with 95 °C for 5 min, followed by 95 °C for 5 s and 58 °C for 5 s. The relative mRNA change rate of the analysis result was analyzed using the 2^−ΔΔct^ calculation method with glyceraldehyde 3-phosphate dehydrogenase (GAPDH) as the reference gene [10].

### 2.5. Oxygen Consumption Rate (OCR)

OCRs were analyzed using the Cell Mito Stress Test Kit (103015-100, Agilent, Santa Clara, CA, USA) in a Seahorse XF96 Pro extracellular flux analyzer (S7850A, Agilent) according to the manufacturer’s instructions. The cells were seeded 5 × 10^4^ cells/well (P4) per XF96 cell culture microplate (103793-100, Agilent) well and further cultured for cell adhesion at 37 °C in a CO_2_ incubator for 24 h. Cortisol treatment was performed by replacing the existing culture media with experimental cortisol-containing culture media according to the treatment time and concentration. As the cortisol treatment was completed, the assay was started after washing with XF assay medium supplemented with 6.2 mM glucose, 1.0 mM sodium pyruvate, and 1.0 mM glutamine, followed by equilibration for 30 min in a non-CO_2_ incubator. The OCR analysis was measured using Oligomycin (1.5 μM/well), FCCP (2.0 μM/well), and Rotenone/Antimycin A (0.5 μM/well). Data were analyzed using Seahorse XF-96 Wave Pro software (version 10.2.0.254) and normalized to each well after obtaining the total protein content using a DC kit (5000113, 5000114, 5000115, Bio Rad, CA, USA). Basal respiration, maximum respiration, ATP production, and spare respiratory capacity were calculated according to the manufacturer’s instructions.

### 2.6. Western Blot

Each protein sample (12 μg) was separated using a 10% polyacrylamide gel and transferred on PVDF membrane, and the washing and blocking process was automatically performed using a Bandmate automated Western Blot Processor (BW1000, Invitrogen). The primary antibody was added to the membrane and incubated overnight at 4 °C. The primary antibody was diluted in 5% skim milk, Caspase-3 (1:2000; bs-0081R, Bioss, Woburn, MA, USA) and GAPDH (1:10,000; MA515738, Invitrogen) antibodies were used. After the primary antibody was removed, the membrane was washed three times for 10 min with TTBS (Tris-Buffered Saline with Tween 20) and the secondary antibody was incubated at room temperature (22–25 °C) for 90 min. The secondary antibody was diluted with 5% skim milk and goat anti-rabbit IgG (1:4000; A16098, Invitrogen) and goat anti-mouse IgG (1:20,000; 311660, Invitrogen) antibodies were used. Protein detection after removal and washing of the secondary antibodies was enhanced using the Pierce ECL Western Blotting Substrate (32106, Thermo Fisher Scientific) and imaged using the iBright CL1000 Imaging System (A32749, Invitrogen). All protein bands were normalized to those of GAPDH and protein expression levels were expressed as relative values for GAPDH. The caspase-3 activity was calculated as the ratio of cleaved caspase-3 to procaspase-3 and was compared with that of the control.

### 2.7. Statistical Analysis of Data

All data analyses were performed using SAS (version 9.4). Data are expressed as mean ± standard error (SEM). All data were analyzed using ANOVA to investigate the effects of cortisol concentration and treatment time. To show statistical significance, all analyses were evaluated on the basis of *p* < 0.05.

## 3. Results

### 3.1. Gene Expression Related to GR Activation Mechanism

**Figure 1:**
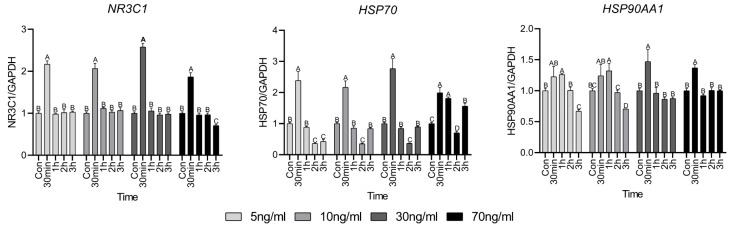
Glucocorticoid receptor activation mechanism-related gene expressions. Values are presented as mean ± standard error. ^A–D^ Different letters in the graph represent significant differences within the same concentration treatment groups (*p* < 0.05).

*NR3C1* encodes the glucocorticoid receptor (GR) and Figure 1 represents the expression level of the gene related to the GR-activated mechanism. *NR3C1* expression was significantly elevated at 30 min of treatment, regardless of cortisol concentration, after which it was similar to that in the control group. However, in the 70 ng/mL group, it was significantly decreased compared to that in the control group at 3 h. *HSP70* and *HSP90AA1* expression was high at 30 min, similar to that of *NR3C1*. However, *HSP70* expression was lower or similar to that in the control after 30 min to 2 h of treatment and a resurgence in expression was observed at 3 h of treatment as the cortisol concentration increased. In contrast, *HSP90AA1* expression gradually decreased after 30 min, regardless of the cortisol concentration, and lower or similar expression was observed compared to the control group.

### 3.2. Cell Metabolism Analysis Results

**Figure 2:**
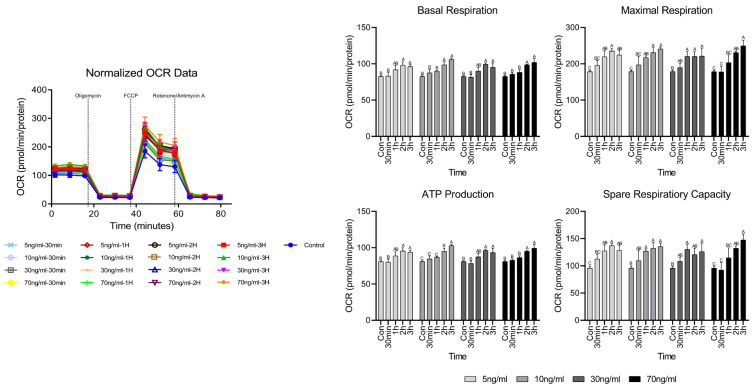
OCR analysis results according to cortisol concentration and treatment time. ^A–C^ Different letters in the graph represent significant differences within the same concentration of treatment (*p* < 0.05).

OCR analysis is a method used to evaluate the respiration function of mitochondria and Figure 2 shows the results of short-term treatment with various cortisol treatment conditions. Evaluating respiratory function, no significant differences were noted in basal respiration and ATP production, compared to the control until 1 h, but both significantly increased after 2 h. Maximum respiration and spare respiratory capacity tended to increase significantly over 30 min.

### 3.3. Gene Expression Related to Antioxidant Factors

**Figure 3:**
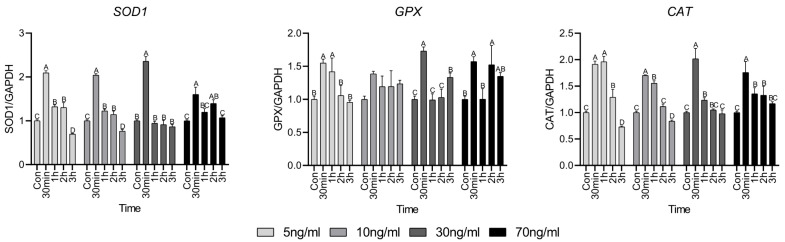
Antioxidant factor-related gene expressions. Values are presented as mean ± standard error. ^A–D^ Different letters in the graph represent significant differences within the same concentration treatment groups (*p* < 0.05).

Figure 3 shows the antioxidant factors used to investigate whether the short-term cortisol treatment has an oxidative effect on HWSC. Antioxidant factors superoxide dismutase 1 (*SOD1*), glutathione peroxidase (*GPX*), and catalase (*CAT*) increased significantly compared to the control group at 30 min and then gradually decreased thereafter. However, in the 70 ng/mL group, all antioxidant factors showed higher expression than in the control, even after 30 min, and especially at 2 h.

### 3.4. Gene Expression Related to Apoptosis Factor

**Figure 4:**
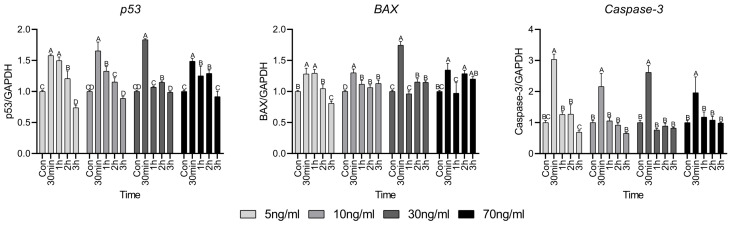
Apoptosis factor-related gene expressions. Values are presented as mean ± standard error. ^A–D^ Different letters in the graph represent significant differences within the same concentration treatment groups (*p* < 0.05).

Cell death factors are closely related to cell survival and the expression of *p53*, *BAX*, and *Caspase-3* is presented in Figure 4. The expression of *p53* was significantly elevated at 30 min, which then gradually decreased over time. However, in the treatment group with concentrations above 30 ng/mL, the expression of *BAX* increased at 2 and 3 h compared to that in the control. Regardless of the cortisol concentration, the expression of *Caspase-3* significantly increased only at 30 min, after which a lower or similar expression was observed compared to the control group.

### 3.5. Expression of Cleaved Caspase-3 and Procaspase-3 Protein

**Figure 5:**
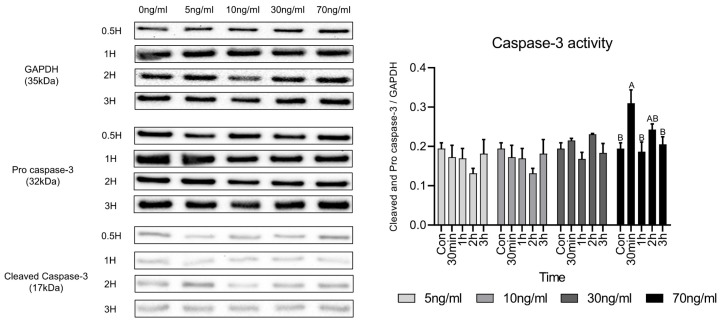
Comparison of caspase-3 activity-related proteins under various cortisol treatment conditions. Values are presented as mean ± standard error. ^A,B^ Different letters in the graph represent statistically significant differences within the same concentration treatment groups (*p* < 0.05).

Caspase-3 activity is caused by the decomposition of procaspase-3 and Figure 5 shows the western blot results to evaluate caspase-3 protein activity following short-term treatment with various concentrations of cortisol. Original images of western blot gels are available in Appendix A.1, Appendix A.2, Appendix A.3 and Appendix A.4. There was no significant difference in the activity of caspase-3 compared to the control at concentrations up to 30 ng/mL. However, in the 70 ng/mL group, there was a significant difference compared with the control. The activity of caspase-3 increased significantly only 30 min after treatment and no significant difference was observed compared to the control after 1 h.

## 4. Discussion

In this study, we investigated the effects of short-term treatment of HWSCs with physiologically detectable levels of cortisol on cell viability, expression of cortisol receptor-operating mechanism genes, antioxidant-related genes, apoptosis genes, protein-related apoptosis activity, and mitochondrial respiration volume.

Cortisol is known to affect cell survival [11]. Common methods for analyzing cell viability include the CCK-8 assay and propidium iodide (PI) staining. The CCK-8 assay assesses cell viability and toxicity by measuring the activity of NADPH-dependent dehydrogenases, which catalyze redox reactions within cells [12]. In addition, the cell count using PI staining was performed by distinguishing the inactive and dead cells; PI dyes were introduced into cells with damaged cell membranes and bound to the genetic material [13]. In this study, when various concentrations of cortisol were administered in the short term, the live cell count did not decrease significantly; however, it is suggested that the CCK-8 result, which decreased with increasing treatment time, had some metabolic effects. Based on these results, the 15-min treatment groups were excluded from the experimental conditions because they were judged to be the basic homeostatic response of cells owing to the instantaneous impact on cells and it was not suitable for investigating the short-term treatment effect of cortisol.

Cortisol is a steroid hormone that directly enters the cell without using transporters or passages in the cell membrane. The GR used to enter the mitochondria is required for the introduction of cortisol to trigger a physiological response [14]. *NR3C1* is one of the major genes involved in GR signaling and its expression pattern can indirectly assess the response of the GR to cortisol [15]. The rapid increase in the expression of *NR3C1* after 30 min of cortisol treatment appears to be a positive feedback response of the GR following the initial inflow of cortisol into the cell, after which it appears to be a negative feedback response. In a study by Sathiyaa and Vijayan [16], when the hepatocytes of trout were exposed to cortisol, *NR3C1* began to increase 24 h after treatment compared to the control. This suggests that cortisol regulates the transcriptional level of GR, showing a trend similar to our study results when treated with cortisol for up to 3 h. However, the cause of the rapid increase in *NR3C1* expression after 30 min is unclear. The ability of cells to respond to glucocorticoids is determined by the number of GR molecules [17] and it seems that after 30 min, this number was sufficient to accommodate cortisol inflow into the cells after rapid *NR3C1* expression occurred. Therefore, a similar trend to the control appears to have emerged. In addition, *HSP70* and *HSP90* involved in GR regulation tended to be different, because the roles involved in GR regulation were different [18].

Cortisol induces intracellular effects on mitochondria and is involved in the overall survival of cells [19]. Many survival mechanisms activated by glucocorticoids, such as cortisol, require additional energy in the form of ATP, which is mainly generated by the oxidative phosphorylation of mitochondria [20]. At this time, reactive oxygen species (ROS) are produced and 0.2% of the consumed oxygen is normally converted to superoxide [21]. A study by Espinoza et al. [22] showed increased ROS activity when cortisol was administered into the myotubes of rainbow trout for 5 min, with maximum activity observed after 15 min. This phenomenon is caused by a non-genomic mechanism against cortisol, indicating a rapid cellular response. You et al. [23] showed that the maximum activity of ROS occurred when mouse hippocampal slices were treated with dexamethasone for 4 h and that GR activation by dexamethasone caused ROS generation. In our study, we indirectly investigated cortisol-induced oxidative stress through OCR analysis of mitochondria heavily influenced by ROS. Basal respiration and ATP production of the cells increased after 1 h of cortisol treatment and maximum respiration and spare respiratory capacity gradually increased as the treatment time continued. During early cortisol treatment, only the maximum respiration increased, whereas basal respiration remained unchanged, suggesting presence of a protective mechanism to preserve mitochondrial integrity under oxidative damage. This subsequent increase in basal respiration may reflect a mitochondrial adaptation to maintain energy balance by producing ATP in response to oxidative damage [24]. Spare respiratory capacity refers to the ability to meet energy demands when the cell is unable to produce sufficient energy. From the results of spare respiratory capacity, it appears that the mitochondria change the amount of metabolism to maintain cell survival in preparation for situations in which they cannot produce energy while continuing cortisol treatment [25]. These results indicate that cortisol increased the ROS activity in cells or mitochondria. If ROS are not properly detoxified under these circumstances, oxidative stress is induced, contributing to the deterioration of function [26]. When oxidative stress is induced, cells are protected from cellular ROS through the induction of the expression of antioxidant factors. SOD transforms the superoxide generated in the mitochondria into a low-reactive form of H_2_O_2_ and CAT and GPX convert it into water and oxygen to protect cells from oxidative stress [27]. The rapid increase in the expression of antioxidant factors compared to the control at 30 min can be seen as an additional generation of antioxidant factors to cope with the accumulation of ROS due to an increase in mitochondrial respiration [28]. However, cortisol treatment for up to 3 h indicated that oxidative stress was not greater than the cellular detoxification capacity of antioxidant factors [29].

Appropriate apoptosis must occur to maintain homeostasis in stressed cells [30]. p53 aids DNA repair by regulating the G1/S regulatory point in the cell cycle and induces apoptosis if the damage is severe [31]. BAX is an apoptosis factor mediated by p53, found in the cytoplasm of normal cells. However, upon receiving an apoptotic signal, it attaches to the membranes of organelles, especially the mitochondrial membrane. This causes the release of cytochrome C and other apoptotic factors from the mitochondria, and finally, apoptosis occurs due to caspase-3 activity [32]. Caspase-3 typically exists in its inactive form as a stable dimer. Upon receiving an apoptotic signal, it is cleaved into two monomers, leading to its activation and ultimately resulting in apoptosis [33]. When cells are exposed to high levels of oxidative stress, the damage can lead to apoptosis or necrosis. Apoptosis, which initially preserves the integrity of the cell membrane, prevents the release of intracellular contents, thereby minimizing inflammation and local tissue damage [34]. Samali et al. [35] showed that menadione induces the production of H_2_O_2_ rather than directly activating caspase-3 and that the resulting H_2_O_2_ subsequently inactivates caspase-3. Pereira et al. [36] showed that the treatment of osteoblasts with cortisol for 14 days slightly reduces the expression of BAX and caspase-3, suggesting that cortisol may inhibit apoptosis. However, because the treatment period was very long, the results have limited applicability to our investigation of short-term cortisol effects. Xu et al. [37] treated mouse hippocampal nerve cells with various concentrations of cortisol for 3 h. A significant difference in cell viability was observed at concentration above 400 µM, suggesting that mitochondrial damage may have induced apoptosis. These findings suggest that short-term cortisol treatment does not significantly impair cell survival and that the cellular response to cortisol is cell-type specific. In this study, the viability of HWSCs was not significantly decreased by the cortisol concentration or treatment duration. From an industrial perspective, this suggests that short-term stress has no significant effect on livestock muscles.

## 5. Conclusions

In the present study, we analyzed the effects of short-term cortisol treatment on HWSCs by evaluating cell viability, gene and protein expression, and mitochondrial respiratory function. Our findings provide fundamental insights into the effects of short-term stress on muscle stem cells and may contribute to the development of countermeasures for stress management in livestock. However, as this study utilized only muscle stem cells derived from Hanwoo, the results may not be directly applicable to other species. Future studies should explore clearer mechanisms by applying cortisol to more complex models, such as coculture, different breeds, and other livestock species.

## Figures and Tables

**Table 1:** CCK-8 results according to cortisol concentration and treatment time.

Concentration	Time							*p* Value
	Con	15 min	30 min	1 h	2 h	3 h	24 h	
Con (0 ng/mL)	100.00 ± 2.27	100.00 ± 2.41 ^A^	100.00 ± 7.25	100.00 ± 3.11	100.00 ± 8.66 ^A^	100.00 ± 5.92 ^A^	100.00 ± 7.62 ^A^	
5 ng/mL	100.00 ± 2.27 ^abc^	90.80 ± 5.48 ^AB, bcd^	102.08 ± 3.35 ^ab^	108.67 ± 5.69 ^a^	78.47 ± 4.30 ^B, d^	85.35 ± 2.43 ^B, d^	87.30 ± 1.14 ^ABC, cd^	0.0003
10 ng/mL	100.00 ± 2.27 ^a^	84.29 ± 6.18 ^BC, bc^	95.97 ± 3.43 ^ab^	95.32 ± 2.64 ^ab^	71.30 ± 6.81 ^B, c^	83.99 ± 1.78 ^B, bc^	74.98 ± 3.23 ^C, c^	<0.0001
20 ng/mL	100.00 ± 2.27 ^a^	73.53 ± 3.12 ^CD, b^	101.89 ± 3.30 ^a^	105.21 ± 6.84 ^a^	70.55 ± 4.39 ^B, b^	72.95 ± 2.80 ^CD, b^	76.49 ± 3.84 ^BC, b^	<0.0001
30 ng/mL	100.00 ± 2.27 ^a^	66.92 ± 1.98 ^D, bc^	104.44 ± 3.57 ^a^	103.01 ± 3.03 ^a^	62.36 ± 3.64 ^B, c^	79.09 ± 2.60 ^BC, b^	73.03 ± 5.89 ^C, bc^	<0.0001
50 ng/mL	100.00 ± 2.27 ^a^	69.60 ± 5.28 ^D, b^	98.80 ± 0.68 ^a^	96.48 ± 4.25 ^a^	78.20 ± 3.44 ^B, b^	69.97 ± 3.51 ^CD, b^	78.89 ± 2.94 ^BC, b^	<0.0001
70 ng/mL	100.00 ± 2.27 ^b^	64.45 ± 3.61 ^D, c^	115.06 ± 2.94 ^a^	92.46 ± 1.64 ^b^	63.24 ± 8.80 ^B, c^	63.26 ± 1.49 ^D, c^	89.92 ± 2.87 ^AB, b^	<0.0001
*p*-value		<0.0001	0.0617	0.1321	0.0024	<0.0001	0.0026	

“^A–D^” indicate the significance when different concentrations of cortisol were treated for the same duration. “^a–d^” indicate the significance when the same concentration of cortisol was treated for different durations.

**Table 2:** Live cell counting results according to cortisol concentration and treatment time.

Concentration	Time						*p* Value
	Con	15 min	30 min	1 h	2 h	3 h	
Con (0 ng/mL)	1.00 ± 0.02	1.00 ± 0.05 ^A^	1.00 ± 0.05	1.00 ± 0.004 ^B^	1.00 ± 0.03	1.00 ± 0.07 ^A^	
5 ng/mL	1.00 ± 0.02 ^ab^	0.79 ± 0.01 ^B, c^	0.93 ± 0.04 ^b^	1.1 ± 0.03 ^A, a^	1.06 ± 0.02 ^a^	0.68 ± 0.02 ^B, c^	<0.0001
10 ng/mL	1.00 ± 0.02 ^ab^	0.71 ± 0.11 ^B, c^	1 ± 0.04 ^ab^	0.9 ± 0.02 ^C, b^	1.13 ± 0.13 ^a^	0.95 ± 0.03 ^A, ab^	0.0034
30 ng/mL	1.00 ± 0.02 ^b^	0.80 ± 0.02 ^B, c^	1 ± 0.04 ^b^	1.11 ± 0.02 ^A, ab^	1.16 ± 0.05 ^a^	1.05 ± 0.01 ^A, ab^	0.0002
70 ng/mL	1.00 ± 0.02 ^ab^	0.68 ± 0.07 ^B, c^	0.99 ± 0.03 ^ab^	1.07 ± 0.03 ^AB, a^	0.89 ± 0.05 ^b^	1.01 ± 0.05 ^A, ab^	<0.0001
*p* value		0.03	0.72	0.0005	0.12	0.007	

“^A–C^” indicate the significance when different concentrations of cortisol were treated for the same duration. “^a–c^” indicate the significance when the same concentration of cortisol was treated for different durations.

**Table 3:** Primer information.

Gene Symbol	Accession Number	Primer Sequences (5′ to 3′)	Product Size (bp)
*NR3C1*	XM_059887932.21	F: ACTCACTGATGGACCCCAAG R: TCTCTCGACCAAGCACACTG	78
*HSP70*	NM_203322.3	F: AGCAGGTGTGTAACCCCATC R: CAGGCAAGACCAAAGTCCAT	181
*HSP90AA1*	NM_001012670.2	F: AGCCCTGAGAGACAACTCCA R: CGTACAGCAGGATGACCAGA	152
*SOD*	NM_174615.2	F: AGAGGCATGTTGGAGACCTG R: CAGCGTTGCCAGTCTTTGTA	189
*CAT*	NM_001035386.2	F: TGGGACCCAACTATCTCCAG R: AAGTGGGTCCTGTGTTCCAG	178
*GPX*	NM_174076.3	F: GGAGATCCTGAATTGCCTGA R: TTAGGGTCGGTCATGAGAGC	174
*p* *53*	NM_174201.2	F: CCTCACCATCATCACACTGG R: GGTAGGCAGTGCTCGCTTAG	178
*BAX*	NM_173894.1	F: CTCCCCGAGAGGTCTTTTTC R: TCGAAGGAAGTCCAATGTCC	176
*Caspase-3*	NM_001077840	F: TCTGGTACAGACGTGGATGC R: CCATGGCTTAGAAGCACACA	173
*GAPDH*	NM_001034034.2	F: GGGTCATCATCTCTGCACCT R: GGTCATAAGTCCCTCCACGA	176

**Figure A1:**
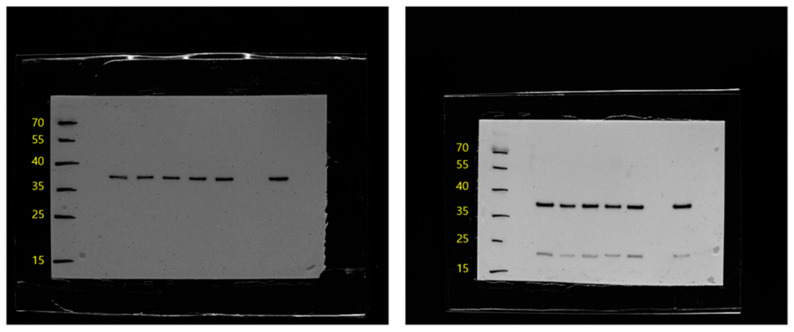
Western blot results of GAPDH and Caspase-3 after 30 min treatment with cortisol at treatment concentrations.

**Figure A2:**
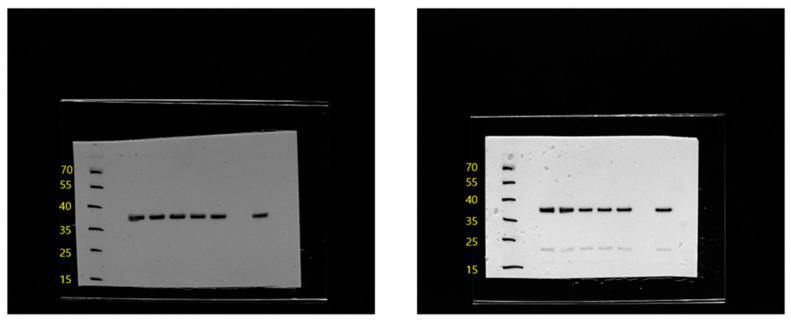
Western blot results of GAPDH and Caspase-3 after 1 h treatment with cortisol at treatment concentrations.

**Figure A3:**
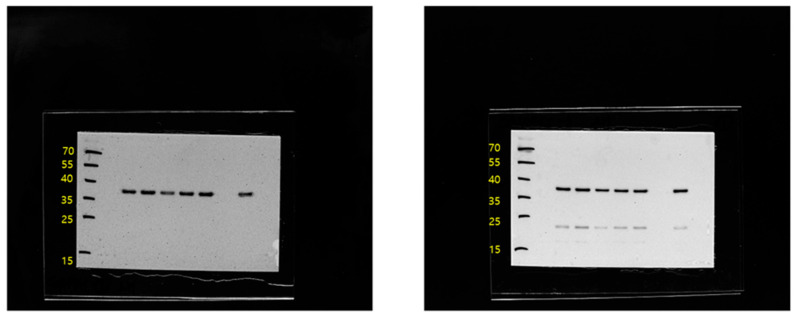
Western blot results of GAPDH and Caspase-3 after 2 h treatment with cortisol at treatment concentrations.

**Figure A4:**
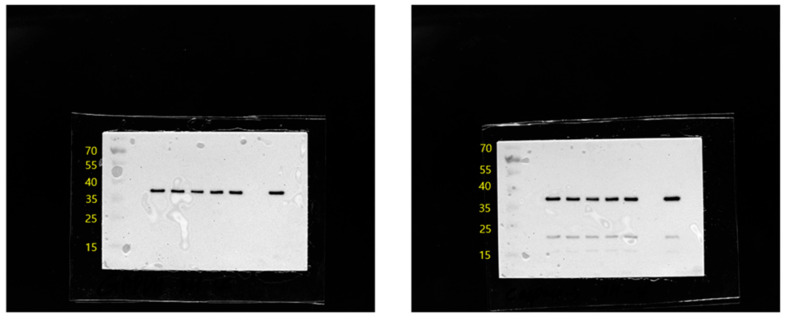
Western blot results of GAPDH and Caspase-3 after 3 h treatment with cortisol at treatment concentrations.

## Data Availability

The dataset used and/or analyzed during the current study is available from the corresponding author on reasonable request.
